# The impact of carbon addition on the organisation of rhizosheath of chickpea

**DOI:** 10.1038/s41598-018-36958-0

**Published:** 2018-12-21

**Authors:** Sheikh M. F. Rabbi, Matthew K. Tighe, Oliver Knox, Iain M. Young

**Affiliations:** 1 School of Life and Environmental Sciences, Centre for Carbon, Water and Food, The University of Sydney, Camden, NSW 2570 Australia; 2 School of Environmental and Rural Science, University of New England, Armidale, NSW 2351 Australia; 3 Faculty of Science, The University of Sydney, Sydney, NSW 2006 Australia

## Abstract

Spatio-temporal development of the rhizosheath during root elongation has the potential to modify the function of the rhizosphere under abiotic stress. We quantified the impact of carbon (i.e. glucose) addition on the development and function of rhizosheath of drought tolerant and sensitive chickpea (*Cicer arietinum* L.) by integrating soil pore volume obtained from X-ray microtomography (µCT), soil physical and microbial respiration measures, and measurements of root traits. Structural equation modelling indicated the feedback mechanisms between added carbon, root traits, pore geometry, and soil functions differed between the cultivars in a fashion congruent with the concept of soil as a self-organising system that interacts with an introduced root system. The drought tolerant cultivar partitioned more photosynthetically fixed carbon to the roots, had more root hairs and more porous rhizosheath, as compared with the sensitive cultivar.

## Introduction

The rhizosphere is the volume of soil that has its function impacted by the root^1,2^. The modification of pore geometry (i.e. changes in porosity, pore connectivity, and/or pore diameter), rhizodeposition (i.e. mucilage, exudates), and root-microbe interactions in the rhizosphere have the potential to modify the hydraulic properties of soil^3–6^. Physical pressure from root growth during cell elongation and root exudation can improve conditions for microbial colonisation, leading to further modification of pore geometry of the rhizosphere by microbes^1,5,7,8^. In addition, the abundance and function of microbes is dependent on the carbon substrate available in the rhizosphere^9,10^, either from rhizodeposition or soil organic matter^11,12^. The quality (e.g. C/N ratio) and quantity of soil organic matter also strongly influences the diversity of organisms present in the soil^10,13,14^. Modification of pore geometry, rhizodeposition and root-microbe interactions in the rhizosphere can also modify water retention and the hydraulic conductivity of the soil^15–17^. This complexity at the soil-root-microbe interface (SRMi) has been described as self-organising by Young & Crawford^18^.

**Figure 1:**
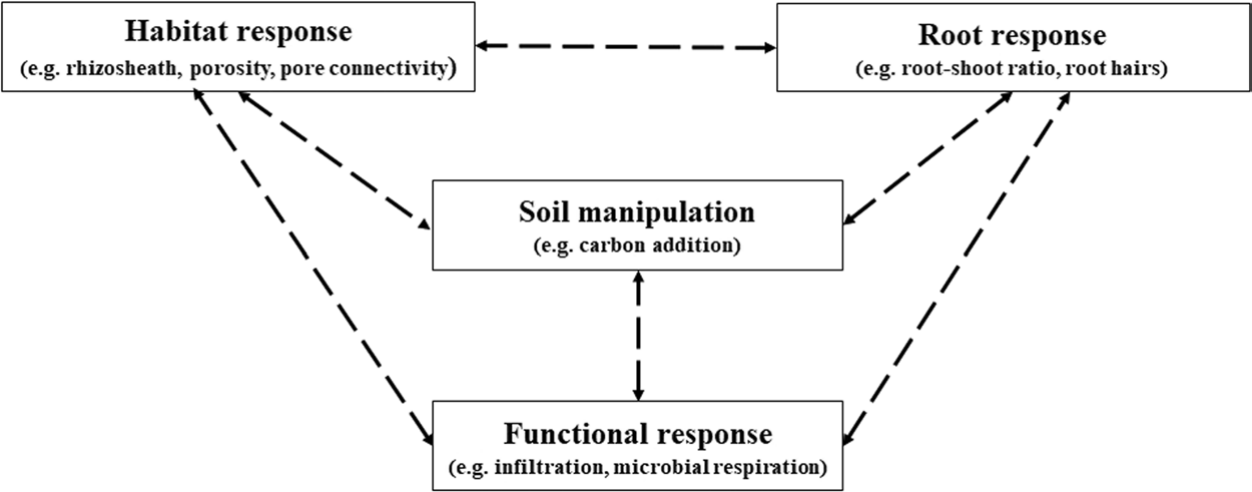
The conceptual model showing links between soil manipulation, root, habitat and functional responses in the rhizosphere.

Thus, there is a complex set of links between the development of the physical rhizosphere and rhizosphere processes that may be strongly influenced by inputs to the system, such as carbon (Fig. 1). Overall, it appears evident that the development of roots can significantly alter the SRMi^5,18,19^. Despite this, it is still unknown how rhizosphere processes interact in the root zone to produce a net benefit in terms of water flow towards the root system. The influence of inputs such as carbon upon these interactions is similarly unknown. However, within the rhizosphere there is a zone of a few millimetres of soil that is closely adhered to the root system, termed the rhizosheath^2^. This is a zone of strong interaction between root exudates and soil habitat, and the development of the rhizosheath itself could be viewed as a precursor to stronger interactions between roots, soil habitat, and microbial activity.

In our previous work, we showed a mechanistic connection between µCT pore geometry and water permeability in the rhizosheath of drought tolerant and sensitive chickpea cultivars^2^. The objective of this work was to investigate the links between pore geometry, root traits and soil functional responses, the association of the rhizosheath with the expected initial development of soil self-organisation, and how the addition of carbon may influence such associations. We use two Chickpea (*Cicer arietinum* L.) cultivars with expected contrasting root traits as model systems, combined with X-ray microtomography (µCT) and soil physical and microbiological measurements.

## Results

### Chickpea root traits, rhizosphere pore geometry and functions

On average, the root-shoot ratio was 15% greater, and root hair area 78% greater in the tolerant cultivar than the sensitive cultivar treatments (P < 0.05). The shoot dry weight was on average 51% greater in sensitive than tolerant cultivar (P < 0.05). The rhizosheath (g soil cm root length^−1^) of the tolerant chickpea was similar to that of sensitive chickpea (Table 1). The µCT porosity of rhizosheath was significantly (~17%) greater than the bulk soil only in the drought tolerant cultivar (P < 0.05). The microbial respiration of rhizosheath soil was significantly greater than bulk soil both in sensitive (~32%) and tolerant (~26%) chickpea (P < 0.05) (Table 2).

**Table 1:** Average values of root-shoot ratio, root hair area, shoot dry weight and rhizosheath soil mass of drought tolerant and sensitive cultivars at no (0 mg C g soil^−1^), moderate (0.5 mg C g soil^−1^) and high (1.5 mg C g soil^−1^) carbon treatments. Means with a different lowercase letter between cultivars or carbon treatments are significantly different (P < 0.05).

Root and shoot traits	Main effects				
	Chickpea		Carbon treatments		
	Sensitive	Tolerant	No carbon	Moderate carbon	High carbon
Root-shoot ratio	0.34a (0.03)	0.39b (0.02)	0.35a (0.04)	0.34a (0.02)	0.41a (0.02)
Root hair area (Proportion of total root area)	0.01a (0.002)	0.02b (0.002)	0.02a (0.005)	0.03a (0.005)	0.01a (0.0005)
Shoot dry weight (mg)	68.50a (6.60)	45.44b (4.53)	63.73a (12.48)	61.24a (12.69)	46.00b (9.42)
Rhizosheath (mg soil cm root length^−1^)	6.20a (0.37)	6.30a (0.51)	5.74a (0.008)	6.39a (0.54)	6.62a (0.70)

The values in the parentheses are standard error of means.

**Table 2:** Average values of porosity, pore connectivity, microbial respiration and water infiltration of rhizosheath and bulk soil of drought sensitive and tolerant chickpeas and unplanted treatment at no (0 mg C g soil^−1^), moderate (0.5 mg C g soil^−1^) and high (1.5 mg C g soil^−1^) carbon treatments.

Habitat and functional responses	Main effects					
	Chickpea	Bulk soil	Rhizosheath	No carbon	Moderate carbon	High carbon
Porosity (%)	Unplanted	—	—	27.23a (1.30)	26.59a (3.19)	25.17a (4.38)
	Sensitive	24.55a (1.32)	25.11a (1.29)	23.41a (0.03)	23.67ab (0.64)	27.40b (0.22)
	Tolerant	23.46a (1.46)	27.49b (1.37)	25.32a (4.63)	24.65a (0.57)	26.46a (0.84)
Pore connectivity	Unplanted	—	—	99.44a (0.14)	99.33a (0.11)	99.07a (0.19)
	Sensitive	80.91a (3.90)	72.57a (4.22)	73.99a (4.06)	84.73a (3.91)	71.50a (4.55)
	Tolerant	89.67a (4.84)	89.77a (6.99)	78.78a (2.74)	98.58b (0.31)	91.80ab (2.59)
Microbial respiration (µg C g^−1^d^−1^)	Unplanted	—	—	4.25a (0.33)	4.46ab (0.18)	5.35b (0.31)
	Sensitive	4.39a (0.12)	5.79b (0.65)	4.73a (0.57)	4.72ac (0.27)	5.82b (1.26)
	Tolerant	4.30a (0.13)	5.43b (0.24)	4.68a (0.51)	4.68a (0.51)	5.23b (0.67)
Water infiltration (cm^3^)	Unplanted	—	—	0.16a (0.004)	0.16a (0.007)	0.17a (0.02)
	Sensitive*	0.17 (0.002)	0.16 (0.03)	0.13 (0.04)	0.19 (0.02)	0.18 (0.004)
	Tolerant*	0.15 (0.03)	0.17 (0.02)	0.20 (0.01)	0.15 (0.05)	0.13 (0.004)

The means of rhizosheath and bulk soil of sensitive and tolerant chickpea are significantly different when the lower case letters are different (P < 0.05). The means of carbon treatments of unplanted, sensitive and tolerant are significantly different when the lower case letters are different (P < 0.05). The values in the parentheses are standard error of means. * Indicates the significant soil × carbon interaction and — indicates not applicable given the experimental design.

While the sensitive cultivar with the high carbon treatment had significantly higher (~16%) µCT porosity than the no and moderate carbon treatments (P < 0.05), the effect of carbon on µCT porosity of the tolerant cultivar and the unplanted treatment overall was not significant (Table 2). The tolerant cultivar had significantly greater (~16%) µCT pore connectivity at moderate carbon compared to other carbon treatments (P < 0.05). The high carbon treatment significantly increased microbial respiration in unplanted (20%, P < 0.05), sensitive (23%, P < 0.01) and tolerant (12%, P < 0.01) treatments compared to no and moderate carbon treatments.

There was a significant interaction between carbon treatment and soil for water infiltration (P < 0.05). Water infiltration in the rhizosheath of tolerant cultivar at moderate carbon was significantly higher than the bulk soil, while water infiltration in rhizosheath of sensitive cultivar was significantly higher than the bulk soil at no carbon (P < 0.05) treatment (Table 2).

### Role of root traits and carbon treatments in rhizosheath formation and function

**Figure 2:**
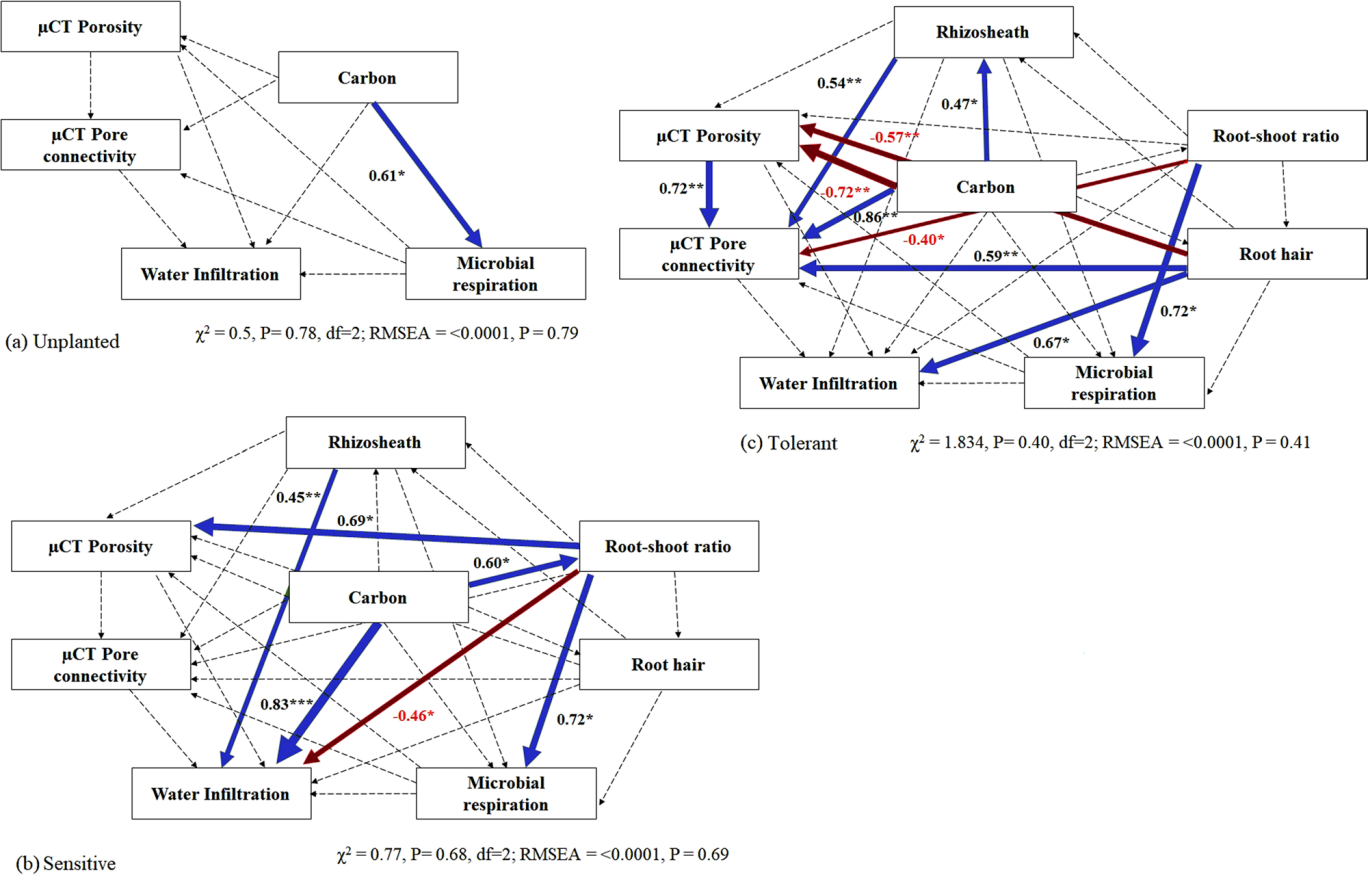
Causal links in SEM of (**a**) unplanted, (**b**) sensitive and (**c**) tolerant cultivars. The numbers adjacent to the arrows represent standardized path coefficients, analogous to regression weights. The width of each arrow is indicative of effect size. Blue arrows indicate significant positive and dark red arrows show negative relationships (*P < 0.05, **P < 0.01, ***P < 0.001). The dashed arrows indicate non-significant relationships. The model fit parameters are shown under each model.

The structural equation modelling indicated that carbon addition had significant positive influence on microbial respiration in unplanted treatments (P < 0.05), but no influence on pore geometry or water infiltration (Fig. 2a). The relationships between added carbon, root traits, pore geometry, infiltration and microbial respiration differed greatly between the tolerant and sensitive cultivars. The sensitive cultivar showed no relationships between carbon additions, rhizosheath and pore geometry (Fig. 2b). Instead, carbon additions significantly increased infiltration (P < 0.001) and root-shoot ratio (P < 0.05). The increase in root-shoot ratio was also associated with an increase in porosity (P < 0.05) and an increase in microbial respiration (P < 0.05). Rhizosheath had positive (P < 0.01), whilst root-shoot ratio had negative (P < 0.05) influence on the water infiltration.

Contrastingly, in the tolerant cultivar, increasing additions of carbon were associated with an increase in rhizosheath (P < 0.05), but not associated with any changes to root-shoot ratio or root hair area (Fig. 2c). The increase in rhizosheath had positive influence on pore connectivity (P < 0.01). Increases of carbon were associated with changes in pore geometry, specifically an increase in pore connectivity (P < 0.001), but a concomitant decrease in porosity (P < 0.01). Carbon additions were not associated with increase in microbial respiration in structural equation model. However, changes in root traits, while appearing independent of carbon addition, were associated with changes in pore geometry, pore connectivity and microbial response. Specifically, increase in root hair area were associated with a decrease in porosity (P < 0.05), an increase in pore connectivity (P < 0.01) and infiltration (P < 0.05), while root-shoot ratio increase were associated with increasing microbial respiration (P < 0.05).

## Discussion

In our previous work, we demonstrated a mechanistic relationship between the modification of pore geometry and water permeability in rhizosheath of drought tolerant and sensitive chickpea cultivars^2^. Unlike our previous work, here we have analysed the pore geometry of the whole root system (both tap and lateral roots) of the chickpea cultivars and investigated the effect of soil carbon on development and function of the rhizosheath.

In the current work, we use SEM to detect and analyse the strength of the relationships among habitat (i.e. porosity, pore connectivity), system manipulation (i.e. carbon treatments), root and functional responses (i.e. microbial respiration, water infiltration) of chickpea cultivars. We also include the development of the rhizosheath as an early indicator of the developing links between soil geometry/habitat and root traits. Comparison of the significant causal links in the SEMs indicate that the root alters the development of the soil’s organisation, and carbon addition influences the habitat-root-functional response linkages, which is strongly influenced by the cultivar type.

Ecologically soil may be defined as a self-organising system^18,20^. The modification of architecture and function of the soil micro-habitat by microbes and active plant root gives rise to a self-organised system^18^. The system has ability to organise physical architecture and activate microbial communities for modifying water transport and nutrient cycling in soil^21^. Crawford *et al*.^19^ showed soil as a self-organised system where microbes significantly modify the pore geometry to manipulate the architecture of the micro-habitat. Here, we examined the influence of system manipulation and the influence of root systems from two distinct cultivars, on the development of the self-organisation of the rhizosheath. The causal links between pore geometry, microbial respiration and water infiltration in the unplanted SEM (Fig. 2a) suggest the origins of the organisation of the system, in that the addition of carbon triggered a microbial response, but this response was not associated with a habitat change. This was expected given the short time frame of this work (i.e. 7 days). Previous work has demonstrated this link between microbial response and pore geometry modification but only over slightly longer time spans (e.g. Crawford *et al*.^19^ using 25 days incubation).

Despite the short time frame of this experiment, the presence of the active chickpea root did appear to trigger or ‘prime’ the self-organisation of the system even in the very early stage of root growth. The causal links in the SEM showed that in the sensitive cultivar, the addition of carbon influenced the root-shoot ratio, which in turn was associated with a change in the pore geometry of the rhizosheath (Fig. 2b). One possible explanation of this is that the addition of carbon may have altered root architecture by influencing cell division processes in the primary root through microbial production of phytohormone like substances^22^.

As well as the links between carbon addition, root traits and habitat, there appeared to be a link between the rhizosheath development and increasing water infiltration. This effect was detected even in the presence of a strong effect of carbon addition on water infiltration. This is most likely an early indication that the priming of the self-organising system by the introduction of the root results in a very early functional response in the soil, and ties in with our previous work^2^. The positive relationship between carbon and water infiltration might be related with the production of hydrophilic substances by microbes in the rhizosheath at high carbon treatment. Watt *et al*.^23^ showed that bacteria can also produce hydrophilic mucilage in the rhizosphere. However, a mature self-organising system exhibits habitat and functional responses of both root and microbes, which was not observed in the rhizosheath of the sensitive cultivar.

Compared to sensitive cultivar, the associations evident in the tolerant chickpea SEM were more complex and could be taken, as a whole, as indicating a stronger self-organisation effect with the introduction of a root system that is more tolerant to water stress (Fig. 2c). This was evident in the general response of the root-shoot ratio and root hair area in this cultivar. As these traits indicated, the tolerant cultivar partitioned more photosynthetically fixed carbon to the root zone. This suggests the tolerant chickpea developed a more complex root system, hence priming the self-organised system response. Root system of the tolerant chickpea had either positive or negative effects on both the habitat architecture and the function (Fig. 2c). The root hair area and root-shoot ratio have direct influence on habitat architecture (through changing root-soil contact^24–26^), infiltration and microbial respiration (possibly through the root exudation of low molecular weight organics and phospholipid type substances^27–29^). The effect of root was decoupled from the addition of carbon, indicating it was a general root trait of the tolerant chickpea cultivar. Similar to the effects of roots on habitat and functions, carbon addition had both negative and positive influences on them. Again, this suggests an early development of a complex self-organising system in which soil particles are reorganising into micro-habitats by microbes with the addition of carbon^30^.

## Conclusion

This work demonstrated that the concept of the self-organisation of soil can be extended to explain the development of rhizosheath of chickpea. It also demonstrated that a simple system manipulation (the addition of a simple carbon source) appears to have more of an effect on soil function and physical habitat development when the root system is tolerant to water stress (i.e. when the soil-root system are more ‘advanced’ in terms of a self-organisation). The extent of rhizosheath development appears to be an early indicator of a complex soil-root system developing, and even after a very short time period of 7 days roots begin to exert influence upon soil functional responses and soil physical habitat. This work has implications for the development of plant cultivars and their responses to stressors and system manipulations, and needs to be extended both temporally and across varieties and plant species. Doing so will increase our understanding of the resilience of the soil self-organisation both natural and managed environments, and how plants may adapt or respond to future stresses, such as water shortages.

## Materials and Methods

### Experimental design

A drought tolerant chickpea (*Cicer arietinum* L.) cultivar (PBA Slasher) and a drought sensitive chickpea cultivar (PBA Hattrick) were grown in 30 mm diameter, 25 cm length polyvinyl chloride (PVC) tubes in oxic soil (sieved <2 mm) with a bulk density of 1.1 Mg m^−3^ (Ferrosol in the Australian Soil Classification^31^). During the experiment the water content was maintained at 80% of field capacity (field capacity of 30%, w/w). Phosphorus (P) and nitrogen (N) were applied at 150 mg P kg^−1^ and 65 mg N kg^−1^ as mono-ammoniumphosphate (NH_4_H_2_PO_4_). Potassium (K) and sulfur (S) were added at the rate of 50 mg K kg^−1^ and 10 mg S kg^−1^ as potassium sulfate (K_2_SO_4_). Carbon (C) as glucose was added to the soil to promote microbial growth/activity at three rates; (no (0 mg C g soil^−1^), moderate (0.5 mg C g soil^−1^), and high (1.5 mg C g soil^−1^)). Pre-germinated seeds of both cultivars were planted approximately 1 cm below the soil surface. There were four replicates of each treatment. To collect enough rhizosheath soil for laboratory analyses, we ran an additional four replicates for the rhizosheath mass analysis. The tubes were kept at 25/15 °C day/night temperature in the glasshouse for seven days. In addition, a plant free control was included (i.e. unplanted) in each of the three carbon treatments (n = 4).

### Pore geometry of the soil and chickpea root system

After 7 days, the chickpea treatments and unplanted tubes were scanned at 5 cm soil depth by X-ray microtomography (µCT) at 160 kV and 200 µA with a voxel resolution of ~16 µm (Phoenix|X-ray, GE Sensing & Inspection, Wunstorf, Germany) to measure the pore geometry (i.e. porosity, pore connectivity) of the soil matrix across all treatment combinations. We acquired 3600 projections with 200 ms timing using a 2000 × 2000 × 1000 detector. The images were reconstructed using Phoenix datos|x reconstruction software (Phoenix|X-ray, GE Sensing & Inspection, Wunstorf, Germany).

### Root and soil pore digital extraction

The chickpea root system of the scanned area was extracted from the image volume using *ROOT1*, a root extraction plug-in for FIJI^32^. Segmentation of the 8 bit grayscale images into pore space was achieved with the FIJI thresholding algorithm^33^.

### 3D Rhizosheath extraction

**Figure 3:**
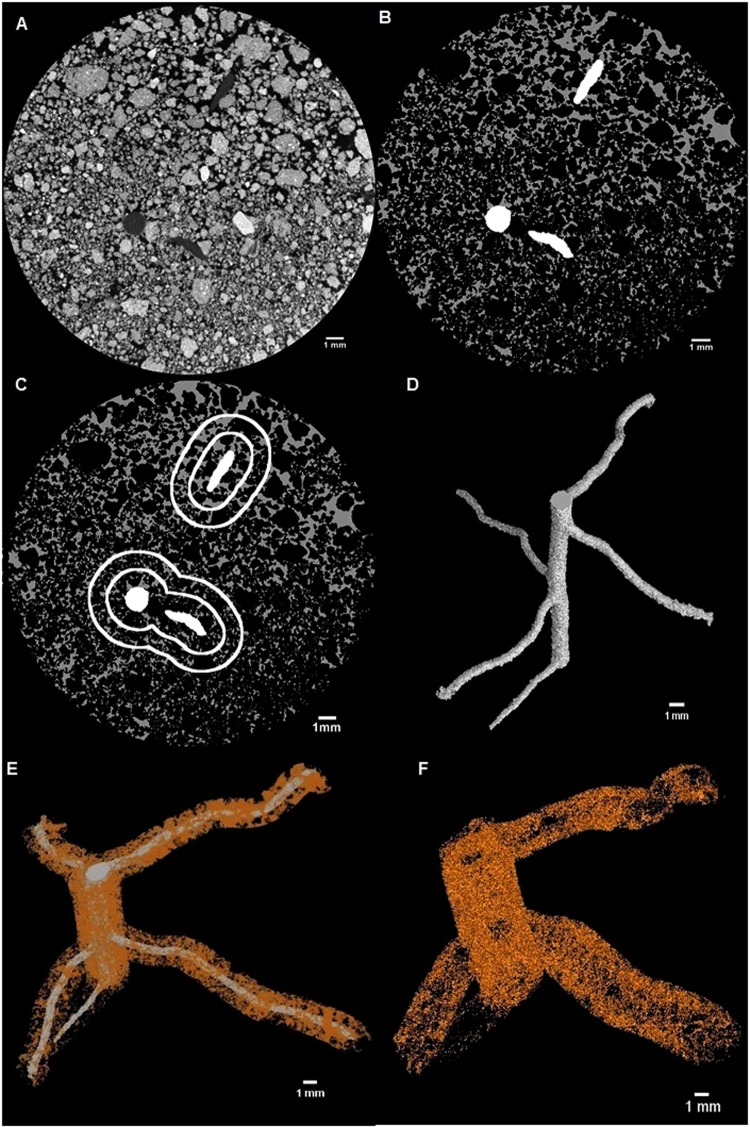
Images showing steps of rhizosheath (0–1 mm) and bulk soil (1–2 mm) from root surface using RHIZOSPHERE macro in Fiji. (**A**) Scanned image with root and soil matrix, (**B**) Segmented pore spaces and root, (**C**) Concentric circle around root to segment pores of rhizosheath and bulk soil, (**D**) Segmented 3D root volume, (**E**) Segmented 3D rhizosheath around root, (**F**) Segmented 3D bulk soil.

**Figure 4:**
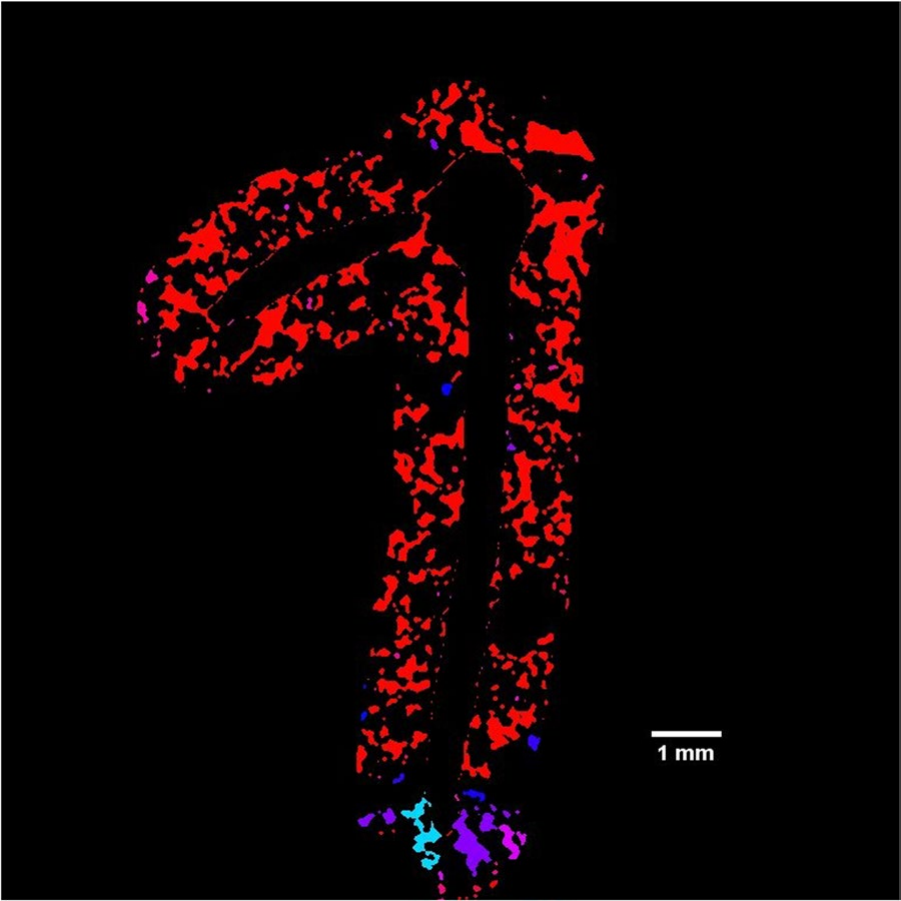
µCT of pore connectivity of the rhizosheath, colours showing different connected regions. Each of the colour represents distinct connected regions.

A change in porosity between rhizosphere and bulk soil was observed in previous works^2,34^. To define the rhizosheath within the 3D µCT image volume, we performed porosity analysis from the root surface of the both chickpea cultivars (denoted as 0) to 6 mm distance in soil with 1 mm increments to detect changes in soil porosity from the root surface towards the bulk soil. We found the 0–1 mm zone (equating to 182 pore voxels per linear mm) had a significantly higher porosity than subsequent increments (P < 0.05), and we defined this as the rhizosheath based on this porosity difference. Other increments did not show any significant differences in porosity. The 0–1 mm rhizosheath from the root surface of primary and lateral roots and 1–2 mm bulk soil were extracted using the custom made *RHIZOSPHERE* macro for FIJI (Fig. 3). The porosity of the extracted rhizosheath and the bulk soil were analysed using the ‘*Analyze Particles’* menu option in FIJI^33^. The porosity that was measured by image analysis is termed ‘µCT porosity’. Since the measurement of pore connectivity (i.e. µCT pore connectivity) of an image stack of 2.5 GB size was computationally prohibitive, the pore connectivity of the middle 100 slices of an image stack was analysed using the “*Find connected regions*” plug-in option in FIJI (Fig. 4). The values of soil µCT porosity and µCT pore connectivity of the unplanted treatment were determined by taking 1 mm soil from the centre of the image stack.

### Destructive extraction of roots and rhizosheath

Following X-ray scanning, the PVC tubes were opened to destructively extract root and rhizosheath material of the tolerant and sensitive chickpea cultivars. The extracted soil column was gently shaken over a plastic tray to remove soil that had not adhered to the root surface. This was considered as the bulk soil. The rhizosheath soil was collected by gently brushing off the soil from the root surface. The collected rhizosheath and bulk soils were oven dried at 40 °C. The roots were washed under running water and preserved in 50% ethanol solution. The extracted root systems were scanned with an Epson V700 flatbed scanner at 1200 dpi and total root length was analysed with WinRhizo® v. 2009c software (Regent Instruments Inc., Quebec, Canada). Total root hair area on the root system was measured after extracting with the FIJI thresholding algorithm^33^. Root and shoot dry matter was dried at 40 °C and weighed.

### Water Infiltration

The dried rhizosheath and bulk soils were packed at a bulk density of 1.1 Mg m^−3^ in a 96 well microplate. The volume of each well was 380 µl. The cumulative water infiltration into the rhizosheath and bulk soil over 30 seconds was measured at −2 cm head using a miniature infiltrometer^35,36^.

### Microbial respiration

Following the method of Campbell *et al*.^37^, approximately 0.5 g each of rhizosheath and bulk soil sample, for each replicate, was packed in 96 deep-well (total well volume 1200 µl) MicroResp® plate. The water content of the soil was maintained at 52% of maximum water holding capacity and the deep-well microplate was pre-incubated at 25 °C in the dark for 3 days. After 3 days a freshly prepared indicator plate was attached with the deep-well microplate and incubated again at 25 °C in dark for 6 hours. The colour change of the indicator plate was measured before and after the incubation using SpectraMax M2^e^ (Molecular Devices, USA) microplate reader at 570 nm^37^. The µg CO_2_-C g soil^−1^ day^−1^ was calculated as described by Campbell *et al*.^37^.

### Statistical analyses

Data was analysed firstly, to summarize and quantify univariate trends, and secondly, to assess the strength of relationships between root responses, habitat responses, functional responses and soil manipulation as outlined in the Fig. 1.

To determine the effect of chickpea cultivars and carbon treatments on root traits (i.e. root-shoot ratio, root hair area, rhizosheath soil mass), two-way analysis of variance (ANOVA) was carried out in R (v 3.4.0)^38^, which allowed the examination of the interaction between chickpea cultivars and carbon treatments. The adequacy of the ANOVA models was assessed by checking model diagnostic plots. Box-Cox transformation was used to normalize the data when necessary using the MASS package, version 7.3–43 in R^39^.

Data was also analysed using two-way ANOVA to determine the effect of carbon treatments on µCT porosity, µCT pore connectivity, microbial respiration and water infiltration of soil (i.e. rhizosheath and bulk soil) within each cultivar with soil × carbon treatments interaction.

One-way ANOVA was performed to compare the effect of carbon treatments on µCT porosity, µCT pore connectivity, microbial respiration and water infiltration of the unplanted treatment. Where appropriate, to compare the means for the levels of a treatment in ANOVA, Tukey Honest Significant Differences (HSD) *post-hoc* test was performed in R. The significant soil × carbon treatment interactions were also analysed using Tukey HSD.

To address the second data analysis objective we performed structural equation modelling using AMOS 24 (IBM SPSS, Amos Development Corporation, Meadville, Pennsylvania, USA) to evaluate the influence of carbon treatments on the root traits, rhizosheath, pore geometry, microbial respiration and water infiltration. To construct a SEM we also hypothesized that root traits, rhizosheath, pore geometry and soil functions has relationship among them (as per Fig. 1). The values of porosity, pore connectivity, water infiltration and microbial respiration in the SEM were the percent changes (either positive or negative) of these traits in rhizosheath compared to bulk soil. As carbon treatment was a categorical variable, we converted the carbon treatments to a set of dummy variables (1–3), to use in SEM^40^. Non-significant chi-square (χ^2^) test, goodness of fit index (GFI) and root mean square error of approximation (RMSEA) were used to find an acceptable SEM model^41,42^.

## Data Availability

Data will be available after acceptance of the manuscript for publication.
